# Secular Trends in the Use of Valproate‐Containing Medicines in Women of Childbearing Age in Europe: A Multinational DARWIN EU Network Study

**DOI:** 10.1002/pds.70232

**Published:** 2025-10-16

**Authors:** Lucía Bellas, Martí Català, Edward Burn, Yuchen Guo, Mike Du, Katia Verhamme, Egil Fridgeirsson, Talita Duarte‐Salles, Tommi Kauko, Eeva Kronqvist, James T. Brash, Sarah Seager, Daniel Prieto‐Alhambra, Annika M. Jödicke, Albert Prats‐Uribe

**Affiliations:** ^1^ Pharmacoepidemiology‐ and Device Group NDORMS, University of Oxford Oxford UK; ^2^ Clinical Pharmacology Department Hospital Universitari Vall d'Hebron Barcelona Spain; ^3^ Department of Pharmacology, Therapeutics and Toxicology Universitat Autònoma de Barcelona Barcelona Spain; ^4^ Department of Medical Informatics Erasmus Medical Center Rotterdam the Netherlands; ^5^ Fundació Institut Universitari per a la recerca a l'Atenció Primària de Salut Jordi Gol i Gurina (IDIAPJGol) Barcelona Spain; ^6^ Auria Clinical Informatics ACI VARHA Turku University Hospital Turku Finland; ^7^ IQVIA, Real World Solutions Brighton UK

**Keywords:** antiepileptic drugs, drug utilization, network study, pharmacoepidemiology

## Abstract

**Background:**

Valproate‐containing medicines (VPA) are first‐line treatments for epilepsy; however, they pose teratogenic risks, restricting their use in women of childbearing age. We aimed to estimate the secular trends in the use of VPA and alternative treatments in young women, and to characterise dose/strength, treatment duration, and indication in new VPA users.

**Methods:**

We conducted a multi‐national population‐based cohort study using primary care records from the Netherlands, Spain, and the UK (IPCI, SIDIAP, CPRD GOLD), primary and outpatient specialist care records from Germany and Belgium (IQVIA DA Germany, IQVIA LPD Belgium), and hospital records from Finland (ACI VARHA), all mapped to the OMOP Common data model. All women present in the databases aged ≥ 12 and ≤ 55 years on the 1st of January of each year in the period 2010–2022 (or latest available), with at least 365 days of prior observation, were included.

**Results:**

A total of 2 948 860 (CPRD GOLD), 718 835 (IPCI), 2 494 052 (SIDIAP), 157 361 (ACI VARHA), 218 250 (IQVIA LPD Belgium); and 5 152 752 (IQVIA DA Germany) women were included. Among those, 6416, 1241, 10 398, 1447, 945, and 4002 started treatments with VPA, respectively. Incidence and prevalence of VPA use in young women decreased between 2010 and 2021, while the prevalence of the alternative treatments pregabalin and gabapentin increased, especially in CPRD (it rises from 0.5% to 1.5%). Median age of new VPA users ranged between 40 and 43 years. Anxiety and depressive disorder were frequent comorbidities, and the use of hormonal contraceptives we were able to capture was low. Average treatment duration varied substantially across databases.

**Conclusion:**

Incidences and prevalence of use of VPA among young women declined since 2015. Conversely, alternative antiepileptics have increased in uptake, particularly gabapentinoids. The use of standardized federated analytics allowed for a rapid assessment of VPA utilization, supporting the regulatory agencies in their decision‐making and improving patient safety across Europe.

**Summary**

- Established Risks of Valproate (VPA): The teratogenic risks associated with VPA are well known, leading to its contraindication in women of childbearing age, with few exceptions to prevent VPA exposure during conception and pregnancy.
- Previous Studies on VPA Use: Prior studies highlighted a reduction in VPA prescriptions following the European Medicines Agency's Risk Minimization Measures (RMM) in 2014 and 2018.
- Study Scope and Methodology: This study, part of the DARWIN EU initiative, used standardized federated analytics to assess VPA use across six European countries, utilizing readily available databases and pre‐tested analytical pipelines.
- Study Findings: The study confirmed a decline in VPA treatment initiation among European women of childbearing age following the implementation of regulatory measures over the last decade.
- Implications: Continued monitoring of VPA utilization is essential to prevent exposure during conception and pregnancy, and the study showcases DARWIN EU's capability to generate rapid, high‐quality evidence for regulatory decision‐making.

**Abbreviations**
ACI VARHA: Auria Clinical Informatics at Turku University Hospital, Wellbeing services county of Southwest Finland—VARHA; formerly, Hospital District of Southwest Finland—HDSF
CDM: common data model
CPRD GOLD: clinical practice research datalink GOLD
DARWIN EU: data analysis and real world interrogation network
DUS: drug utilisation study
EHR: electronic health records
EMA: European medicines agency
GP: general practitioner
ICPI: integrated primary care information project
OMOP: observational medical outcomes partnership
PRAC: pharmacovigilance risk assesment commmitee
RMM: risk minimisation measures
SIDIAP: Sistema d'Informació per al Desenvolupamen de la Investigació en Atenció Primària
VPA: valproate acid valproate‐containing medicine

## 1 Background

Valproate and valproate‐containing medicines (VPA) are authorised for the treatment of epilepsy, migraine prophylaxis, and for acute mania in patients with bipolar disorder in Europe [1]. The teratogenic risks associated with the use of valproate in pregnant women are well established [2, 3]: prenatal exposure to valproate substantially increases the risk of neurodevelopmental impairment and congenital malformations in the unborn child. Therefore, its use in women of childbearing age is contraindicated, with few exceptions, to reduce the risk of VPA exposure during conception and pregnancy.

In October 2014, the European Medicines Agency (EMA) Pharmacovigilance Risk Assessment Committee (PRAC) reinforced warnings on VPA use among women of childbearing age. These recommendations emphasised that valproate and related substances should not be used in girls, women of childbearing potential, and pregnant women unless alternative treatments are ineffective or not tolerated. Specifically, they should be contraindicated in the prophylaxis of migraine attacks in pregnancy and in women of childbearing potential who are not using effective methods of contraception [4]. However, a review by the EMA's Pharmacovigilance Risk Assessment Committee (PRAC) in 2018 showed that despite the 2014 Risk Minimization Measures, many patients were still not well informed about the risks of VPA exposure during pregnancy [5]. Studies showed a high level of VPA use among women of childbearing age and pregnant women, suggesting that prescribing conditions were not sufficiently adhered to [7, 8, 9, 10]. In March 2018, the PRAC recommended an update of the RMMs, including the introduction of a comprehensive pregnancy prevention programme [11]. Recently, additional guidelines and restrictions have been issued to minimise VPA use in both men and women aged < 55, owing to the significant risk of serious harm to the baby if taken during pregnancy [12, 13, 14, 15]. The aforementioned findings underscore the abundance of regulatory efforts and the crucial role of electronic healthcare databases in analysing their impact on prescription patterns in specific populations. Therefore, as an initial pilot study, the EMA requested a Drug Utilisation Study as the very first such analysis conducted within the framework of the Data Analysis and Real‐World Interrogation Network (DARWIN EU). DARWIN EU aims to generate actionable rapid evidence on the use, safety, and effectiveness of medicinal products in Europe. First, we analysed the secular trends in the incidence and prevalence of the use of VPA in women of childbearing age in 6 European countries. Secondly, we characterised new users of VPA, including their indication, initial dose, and duration of the new treatment.

## 2 Methods

### 2.1 Data Sources

This network cohort study was conducted using routinely collected healthcare data from six databases from six countries across Europe. We included primary care data from the Netherlands, Spain, and the United Kingdom (ICPI [Integrated Primary Care Information Project], SIDIAP [15, 16] [Sistema d'Informació per al Desenvolupament de la Investigació en Atenció Primària], and CPRD GOLD [Clinical Practice Research Datalink]); primary care and outpatient specialist data from Belgium and Germany (IQVIA LPD Belgium [Longitudinal Patient Database] and IQVIA DA Germany [Disease Analyzer Germany]); and hospital records from one large hospital in South Finland (ACI VARHA [Auria Clinical Informatics at Turku University Hospital]).

All databases were previously mapped to the OMOP (Observational Medical Outcomes Partnership) common data model (CDM) and onboarded as data partners to the DARWIN EU Network. This enabled the use of standardised analytics and tools developed for DARWIN EU across the network since the structure of the data and the terminology system were harmonised.

A detailed description of the contributing data sources is provided in Table 1.

**TABLE 1:** Database characteristics.

Country	Database	Setting	Population ‐ approximate coverage of the population (%)	Last available data	Drug information
Netherlands	IPCI	Primary care	2.82 million‐ (~78%)	2021	Prescription records
Spain	SIDIAP		8.5 million (~74%)	2021	Prescription and dispensation records
UK	CPRD GOLD		17.3 million (~20%)	2019	Prescription records
Belgium	IQVIA LPD Belgium	Primary care and outpatient specialist care	1.13 million (~10%)	2022	GP prescriptions
Germany	IQVIA DA Germany		42 million (~42.5%)	2022	Prescriptions of marketed products
Finland	ACI VARHA	In and out‐patient specialist care	820 000 (~15%)	2021	Inpatient and outpatient specialist prescriptions. Information on drug dose and duration not available for inpatient prescriptions (> 70% of prescriptions).

Abbreviations: ACI VARHA, Auria Clinical Informatics at Turku University Hospital; CPRD GOLD, Clinical Practice Research Datalink; DA, Disease Analyzer; ICPI, Integrated Primary Care Information Project; LPD, Longitudinal Patient Database; SIDIAP, Sistema d'Informació per al Desenvolupament de la Investigació en Atenció Primària; UK, United Kingdom.

### 2.2 Study Design and Study Population

All women aged between ≥ 12 years and ≤ 55 years on 1st of January of each year in the period 2010–2022 (or the latest data available), with at least 365 days of data availability, became eligible and comprised the source population.

To calculate the prevalence denominator population, users contribute person‐time starting from the latest of the following dates: the study start date (January 1, 2010), the date they have at least 1 year of recorded medical history, or the date they reach the minimum age (12 years for the overall population or the lower age limit for age‐stratified groups). They stop contributing person‐time at the earliest of the following dates: the study end date (when data is no longer available in each source), the end of their observation period (meaning loss to follow‐up, end of data availability, death), or the last day they remain within the maximum age (55 years for the overall population or the upper age limit for age‐stratified groups). For prevalence rate calculation, participants were followed up from the day of therapy initiation, that is, the date of the first prescription of valproic acid/valproate‐containing medication (index date), until the earliest of loss to follow‐up, end of data availability, death, or end of continuous exposure.

For incidence calculations, additional criteria were applied: To calculate the incidence denominator, we applied a 365‐day washout since the day of discontinuation. Prescriptions of VPA were identified from RxNorm codes in the mapped data. VPA included valproic acid, sodium valproate, magnesium valproate, valproate semisodium, and valpromide. The quality of the recording of VPA was assessed using the DrugExposureDiagnostics package [17] DrugExposureDiagnostics. While detailed outputs are not publicly shared due to data sensitivity, all tools are open‐source, and results were reviewed internally by data partners and the DARWIN‐EU Coordination Centre.

### 2.3 Statistical Analyses

All analyses were conducted separately for each database using a common analytical script developed by the study coordinators and run locally by each data partner. Only aggregated results were shared. The study code is available on Github (DrugUtilisation). The study was registered in the HMA‐EMA Catalogue of RWD studies (EUPAS50789), and the full study protocol is published. All analyses were conducted in R (version 4.2.1) using the IncidencePrevalence [18] and DrugUtilisation [19] packages. Cell counts < 5 were suppressed for privacy protection.

### 2.4 Population‐Level VPA Utilisation and Alternative Treatments

Prevalence was calculated as annual period prevalence, which summarised the total number of individuals who used the drug of interest during a given year divided by the population at risk of getting exposed during that year. Therefore, period prevalence gave the proportion of individuals exposed at any time during a specified interval. 95% confidence intervals were calculated using the Wilson Score method for binomial distribution.

Annual incidence rates were estimated as the number of *new drug users* divided by the accumulated person‐time of the population at risk of getting exposed during the period for each calendar year, and the result was provided as the number of new users per 100,000 person‐years. 95% confidence intervals were calculated using the exact method for the Poisson distribution.

### 2.5 Summary Patient Characteristics and Drug Utilisation

Summary patient characteristics, including baseline demographics, comorbidities, and comedication, were calculated. Regarding comorbidities, these were chosen comprising a broad range of relevant conditions, which were previously specified for a proper characterisation. We also did large‐scale characterisation, where all conditions recorded in VPA patients' history in the respective databases are registered (https://data‐dev.darwin‐eu.org/EUPAS50789/). A single outpatient diagnosis was considered sufficient for the identification of a comorbidity in this study. These comorbidities were evaluated in three time intervals relative to the index date (ID): between 30 and 1 day before the ID, between 365 and 31 days before the ID, and at any time prior to the ID.

Regarding valproate and alternative treatments, we considered alternative medications, addressing other indications for valproic acid (VPA). Treatments for epilepsy, bipolar disorder, and migraine prophylaxis were examined. These alternatives included other antiepileptics, selected antipsychotics, anxiolytics, amitriptyline, flunarizine, specific beta‐blockers, as well as pizotifen and clonidine (Table 2). We estimated the use of systemic hormonal contraceptives in the year preceding VPA initiation by database and age group.

**TABLE 2:** Drugs of interest and alternative treatments for the same indications (Population‐level Utilisation study only).

ATC code	Name
N03AG01	Valproate
N03AG02	Valpromide
N03AF01	Carbamazepine
N03AA02	Phenobarbital
N03AB02	Phenytoin
N03AA03	Primidone
N05BA09	Clobazam
N03AE01	Clonazepam
N03AF04	Eslicarbazepine acetate
N03AX09	Lamotrigine
N03AF02	Oxcarbazepine
N03AX22	Perampanel
N03AF03	Rufinamide
N03AX11	Topiramate
N03AX15	Zonisamide
N03AX23	Brivaracetam
N03AD01	Ethosuximide
N03AX12	Gabapentin
N03AX18	Lacosamide
N03AX14	Levetiracetam
N03AX16	Pregabalin
N03AG06	Tiagabine
N03AG04	Vigabatrin
N05AN01	Lithium
N05AH04	Quetiapine
N05AH03	Olanzapine
N03AX09	Lamotrigine
C07AA05	Propranolol
C07AB02	Metoprolol
C07AB03	Atenolol
C07AA12	Nadolol
C07AA06	Timolol
C07AB07	Bisoprolol
N03AX11	Topiramate
N06AA09	Amitriptyline
N07CA03	Flunarizine
N02CX01	Pizotifen
N02CX02	Clonidine
G03A	Sex hormones and modulators of the genital system

For each new valproate (VPA) prescription, the initial prescribed dose or product strength was retrieved from the drug_exposure and drug_strength tables of the OMOP CDM, where available. This information was summarized using the minimum, 25th percentile (p25), median, 75th percentile (p75), and maximum dose/strength. The quality and completeness of dose and strength recording varied across databases. In some data sources (e.g., SIDIAP, IPCI), dose, quantity, and duration were explicitly recorded. In others (e.g., CPRD, IQVIA DA Germany), only the strength of the product was available, without direct information on the daily dose or duration. In these cases, dose was approximated based on the product strength and standard therapeutic regimens, including WHO‐defined Defined Daily Doses (DDD) or national clinical guidelines (e.g., 600–750 mg/day for epilepsy or bipolar disorder, 500 mg/day for migraine).

Treatment duration was calculated as the length of the first continuous exposure episode, defined as the prescription end date minus the start date plus 1 day. When explicit dates were unavailable, duration was estimated from the quantity dispensed and the assumed daily dose (DDD). Consecutive prescriptions with gaps of less than 30 days were concatenated into continuous treatment episodes. For ACI VARHA, dose and duration information was only available for outpatient prescriptions, as inpatient records (> 70% of prescriptions) lacked this information.

A proxy was used to retrieve indications: For each prescription, we assessed whether a diagnosis code of any of the respective indications (specific: epilepsy, bipolar disorder, migraine prevention; “any other diagnoses”) was recorded at the date of the VPA prescription, or in the week or month before. If a person had a record of more than one specific indication, that person was included in both specific indication groups separately.

### 2.6 Role of the Funding Source

The study sponsor (EMA/EMRN) requested a drug utilization study for VPA. This study was funded by EMA and performed via DARWIN EU. The lead investigators (APU and AMJ) designed the study using the methods defined in the DARWIN EU Catalogue of Standard Analytics. EMA reviewed and approved the study protocol. EMA had no role in the collection, analysis, and interpretation of data. The full study report was reviewed and approved by EMA and is publicly available, and EMA reviewed the manuscript prior to publication.

## 3 Findings

Results from all drug utilisation analyses including stratifications are available in the interactive web application (https://data‐dev.darwin‐eu.org/EUPAS50789/).

### 3.1 Study Population

A total of 2 948 860 (CPRD GOLD), 718 835 (IPCI), 2 494 052 (SIDIAP), 157 361 (ACI VARHA), 218 250 (IQVIA LPD Belgium); and 5 152 752 (IQVIA DA Germany) women were included. Among those 6416, 1241, 10 398, 1447, 945, and 4002 started treatments with VPA during the study period, respectively.

### 3.2 Population‐Level VPA Utilisation and Alternative Treatments

**FIGURE 1:**
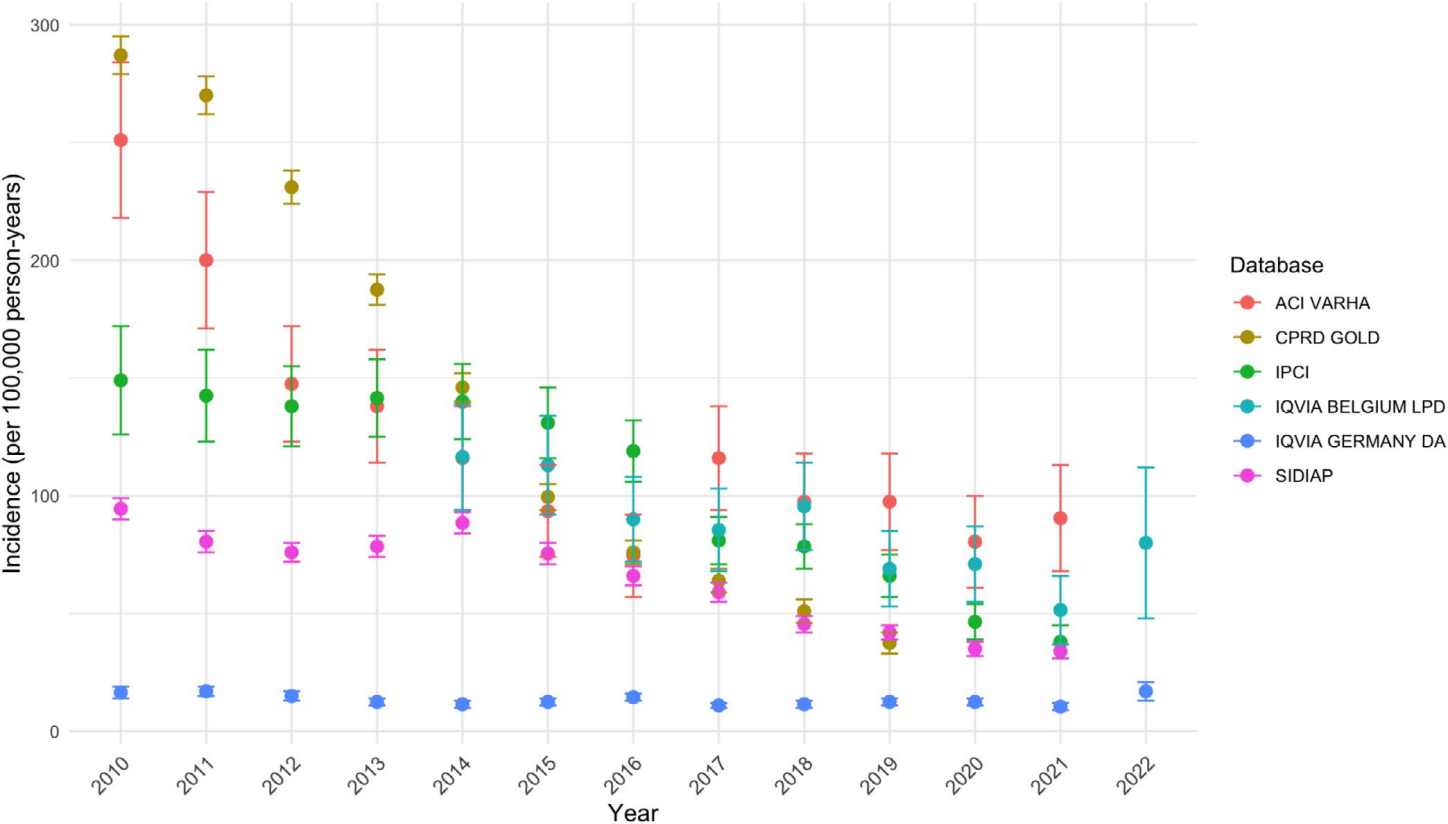
Annual incidence rates of VPA use in women aged between 12 and 55 years in 2010–2022.

The incidence of new use of VPA amongst young women decreased over the period 2010–2021 for all databases, except for IQVIA DA Germany, where incidence rates were stable over time (Figure 1). The sharpest decrease was seen in CPRD GOLD, with the number of new prescriptions per 100,000 person‐years decreasing from 287 (95% CI 279 to 295) in 2010 to 37 (33 to 42) in 2019.

**FIGURE 2:**
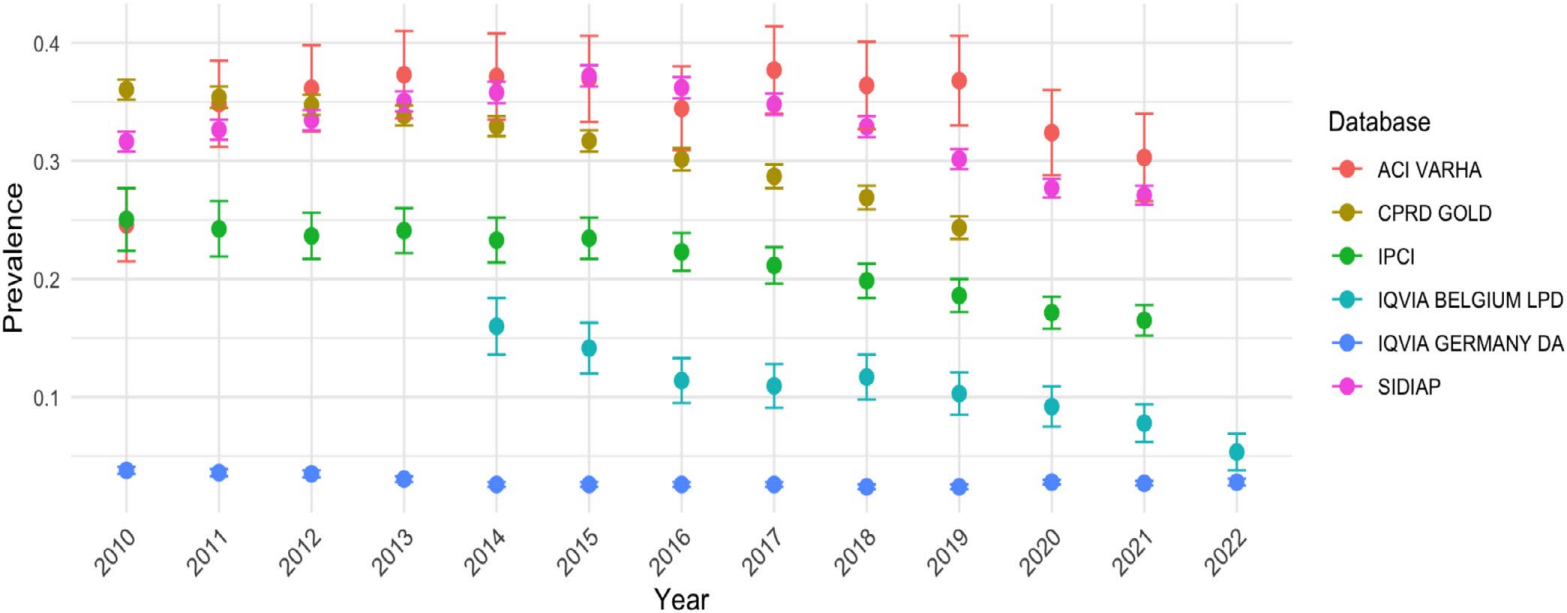
Prevalence of VPA use in women aged between 12 and 55 years in 2010–2022.

Similar to the incidence rates of VPA use, prevalence decreased over the study period for all databases; however, this decrease was less pronounced for ACI VARHA and IQVIA DA Germany. Figure 2 illustrates the trends of VPA prevalence over time for all databases. Prevalence was higher in the older compared to younger age groups in all datasets, and a more pronounced decrease in VPA use was seen in younger age groups after 2015.

Regarding incidence and prevalence stratified by age, the incident use of VPA decreased during the study period for all age groups. In ACI VARHA, IPCI, IQVIA Belgium, and IQVIA Germany all age groups show a decrease in incidence of VPA, especially in the younger age groups, however the sample size is too small to observe any meaningful differences in patterns by age. In what refers to prevalence, in all datasets the prevalence of use of VPA increased with age. The older age groups (≥ 45 or more—55 years old) had prevalences of use between 0.4% and 0.5% and remained stable or increased during the study period. Conversely, younger age groups (< 45) had a lower prevalence, and which decreased over time, halving the initial prevalence at the end of the study for most databases. Incidence of use of VPA showed a decreasing pattern for all age groups in all database.

**FIGURE 3:**
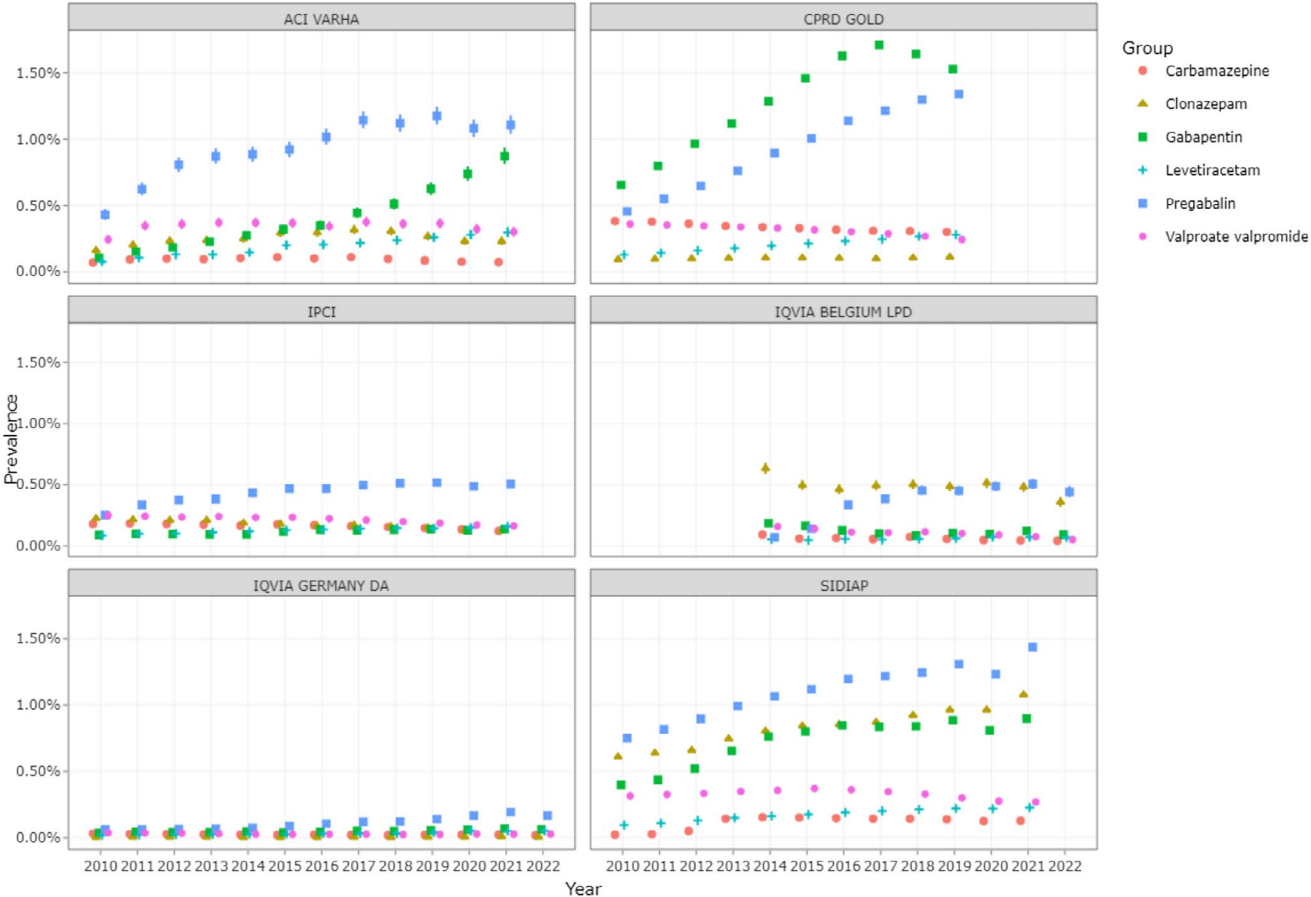
Prevalence of VPA and the 5 most used alternative treatments in women aged between 12 and 55 years in 2010–2022.

Figure 3 shows the prevalence of alternative treatments. Overall, the most prevalent alternative treatments used were pregabalin (rising from 0.5% to 1.5% in CPRD, ACI VAHRA, and SIDIAP and remaining stable in the other databases), gabapentin (which shows the same pattern as pregabalin), clonazepam, carbamazepine, and levetiracetam (the three of them show stable prevalences around 0.5% in all databases). The prevalence of the use of pregabalin and gabapentin clearly increased during the study period, while the others followed different trends across the participating databases.

### 3.3 Patient Characteristics and Drug Utilisation

Table 3 summarises the characteristics of all women at the time of their first prescription of VPA during the study period. Patient‐level drug utilisation was not conducted in ACI VARHA as only limited information on VPA products was available. The median age of new users of VPA ranged between 40 and 43 years. Anxiety and depressive disorder were frequent comorbidities, with 20%–39% and 16%–44% having a record of these respectively at any time before treatment initiation. The prevalence of prescribed contraceptives was highest in CPRD GOLD, followed by IQVIA Belgium LPD, IPCI, and lowest in SIDIAP and IQVIA Germany DA. The captured use of hormonal contraception varied greatly across age groups, with the highest levels of prescriptions observed in women aged 15–39 and lower rates in the > 50‐ and 12–14‐year‐olds (Table 4).

**TABLE 3:** Summary baseline characteristics of new VPA users.

Database	CPRD GOLD	IPCI	SIDIAP	IQVIA belgium LPD	IQVIA germany DA
Number subjects	6416	1241	10 398	945	4002
Age					
Median [p75–p75]	40 [29–47]	43 [32–49]	40 [30–48]	41 [31–49]	43 [31–50]
Visit occurrence (prior year)					
Median [p75–p75]	29 [17–44]	7 [4–12]	14 [7–24]	6 [3–10]	5 [2–11]
General conditions (any time prior)					
Anxiety	2260 (35.2%)	392 (31.6%)	4099 (39.4%)	307 (32.5%)	806 (20.1%)
Asthma	1017 (15.9%)	102 (8.2%)	545 (5.2%)	143 (15.1%)	234 (5.8%)
Chronic kidney disease	146 (2.3%)	< 5	128 (1.2%)	< 5	63 (1.6%)
COPD	82 (1.3%)	21 (1.7%)	96 (0.9%)	113 (12%)	135 (3.4%)
Dementia	23 (0.4%)	< 5	37 (0.4%)	< 5	64 (1.6%)
Depressive disorder	2460 (38.3%)	194 (15.6%)	2610 (25.1%)	414 (43.8%)	1420 (35.5%)
Diabetes	252 (3.9%)	57 (4.6%)	366 (3.5%)	59 (6.2%)	208 (5.2%)
GERD	174 (2.7%)	19 (1.5%)	262 (2.5%)	172 (18.2%)	84 (2.1%)
Heart failure	13 (0.2%)	6 (0.5%)	18 (0.2%)	< 5	43 (1.1%)
Hypertension	333 (5.2%)	96 (7.7%)	601 (5.8%)	166 (17.6%)	431 (10.8%)
Hypothyroidism	366 (5.7%)	56 (4.5%)	896 (8.6%)	104 (11%)	313 (7.8%)
Infertility	63 (1%)	NA	144 (1.4%)	< 5	< 5
IBD	40 (0.6%)	6 (0.5%)	36 (0.3%)	7 (0.7%)	30 (0.7%)
Malignant neoplastic disease	199 (3.1%)	59 (4.8%)	332 (3.2%)	27 (2.9%)	137 (3.4%)
Osteoporosis	44 (0.7%)	7 (0.6%)	84 (0.8%)	22 (2.3%)	32 (0.8%)
Pneumonia	89 (1.4%)	51 (4.1%)	369 (3.5%)	29 (3.1%)	115 (2.9%)
Rheumatoid Arthritis	25 (0.4%)	9 (0.7%)	24 (0.2%)	5 (0.5%)	32 (0.8%)
Stroke	81 (1.3%)	37 (3%)	142 (1.4%)	14 (1.5%)	81 (2%)
Venous thromboembolism	88 (1.4%)	25 (2%)	59 (0.6%)	28 (3%)	65 (1.6%)
Medications (prior year)					
Agents acting on RAAS	366 (5.7%)	132 (10.6%)	618 (5.9%)	59 (6.2%)	228 (5.7%)
Antibacterials (systemic)	3043 (47.4%)	395 (31.8%)	3668 (35.3%)	308 (32.6%)	441 (11%)
Antidepressants	3774 (58.8%)	381 (30.7%)	6243 (60%)	365 (38.6%)	1102 (27.5%)
Anti‐inflammatory and antirheumatic agents	2233 (34.8%)	460 (37.1%)	5357 (51.5%)	368 (38.9%)	627 (15.7%)
Antineoplastic agents	< 5	18 (1.5%)	100 (1%)	11 (1.2%)	18 (0.4%)
Antithrombotic drugs	448 (7%)	78 (6.3%)	263 (2.5%)	27 (2.9%)	101 (2.5%)
Beta Blocking Agents	1079 (16.8%)	242 (19.5%)	695 (6.7%)	167 (17.7%)	270 (6.7%)
CCB	220 (3.4%)	50 (4%)	219 (2.1%)	30 (3.2%)	86 (2.1%)
Diuretics	314 (4.9%)	56 (4.5%)	348 (3.3%)	30 (3.2%)	172 (4.3%)
Drugs for Acid related disorder	1861 (29%)	382 (30.8%)	3519 (33.8%)	258 (27.3%)	507 (12.7%)
Drugs for obstructive airway diseases	1242 (19.4%)	315 (25.4%)	1858 (17.9%)	207 (21.9%)	197 (4.9%)
Drugs used in diabetes	262 (4.1%)	45 (3.6%)	283 (2.7%)	47 (5%)	102 (2.5%)
Immunosuppressants	48 (0.7%)	12 (1%)	76 (0.7%)	< 5	18 (0.4%)
Lipid modifying agents	414 (6.5%)	82 (6.6%)	736 (7.1%)	63 (6.7%)	97 (2.4%)
Opioids	2149 (33.5%)	180 (14.5%)	1289 (12.4%)	206 (21.8%)	183 (4.6%)
Psycholeptics	3484 (54.3%)	626 (50.4%)	8030 (77.2%)	432 (45.7%)	1453 (36.3%)
Psychostimulants	26 (0.4%)	36 (2.9%)	429 (4.1%)	6 (0.6%)	37 (0.9%)

Abbreviations: CCB, Calcium channel blockers; COPD, Chronic Obstructive Pulmonary Disease; GERD, Gastroesophageal Reflux Disease; IBD, Inflammatory Bowel Disease; RAAS, renin‐Angiotensin‐Aldosteron‐System.

**TABLE 4:** Use of systemic, hormonal contraceptives in women starting VPA treatment by database and age.

Database	CPRD GOLD	IPCI	SIDIAP	IQVIA LPD belgium	IQVIA DA germany
General use	1291 (21.9%)	136 (11%)	415 (4%)	150 (15.9%)	59 (1.5%)
Age group (years)					
12–14	< 5	< 5	< 5	< 5	0
15–19	103 (28.8%)	7 (10.6%)	42 (5.4%)	10 (16.7%)	< 5
20–24	227 (42.8%)	14 (15.4%)	65 (10.2%)	19 (27.9%)	10 (3.8%)
25–29	239 (39.1%)	14 (16.3%)	42 (5.7%)	13 (19.4%)	10 (2.8%)
30–34	227 (32.4%)	11 (11.6%)	73 (6.8%)	26 (29.2%)	< 5
35–39	223 (27.3%)	23 (16.8%)	66 (4.6%)	20 (16.7%)	7 (1.7%)
40–44	146 (14.8%)	21 (10.7%)	69 (4%)	24 (18.2%)	< 5
45–49	80 (7.2%)	28 (11.6%)	35 (2%)	29 (17.2%)	14 (2%)
50–54	41 (4.2%)	16 (6.4%)	18 (1.1%)	8 (4.3%)	7 (0.8%)
55	< 5	< 5	< 5	< 5	< 5

On the date of VPA therapy initiation, most women had no record of any of the three pre‐specified indications in all databases except for IQVIA Belgium LPD (Table 5). Among those with a specific diagnosis recorded, on prescription day, migraine was the most common recorded indication (5.7%) in CPRD GOLD, whereas epilepsy was most common in all other databases (IPCI: 8.1%, SIDIAP: 2.6%, IQVIA Belgium: 40.4% and IQVIA Germany: 25.1%). Notably, VPA for migraine was uncommon in SIDIAP. Sensitivity analyses assessing indications within 7‐, 30 days or anytime prior to VPA initiation showed that epilepsy was the most common indication in all databases.

**TABLE 5:** Pre‐specified indications at date of first prescription, and within 7, 30 days, and anytime before for all age groups during the whole study period.

Database	CPRD GOLD	IPCI	SIDIAP	IQVIA LPD belgium	IQVIA DA germany
Number of subjects	6416	1241	10 398	945	4002
At prescription day					
No indication^a^	1961 (30.6%)	802 (64.6%)	8207 (78.9%)	28 (3%)	1472 (36.8%)
Unknown indication^b^	3661 (57.1%)	239 (19.3%)	1699 (16.3%)	227 (24%)	1192 (29.8%)
Bipolar disorder	82 (1.3%)	9 (0.7%)	189 (1.8%)	68 (7.2%)	184 (4.6%)
Epilepsy	350 (5.5%)	101 (8.1%)	270 (2.6%)	382 (40.4%)	1006 (25.1%)
Migraine	363 (5.7%)	90 (7.3%)	34 (0.3%)	286 (30.3%)	174 (4.3%)
7 days					
No indication^a^	935 (14.6%)	683 (55%)	7520 (72.3%)	28 (3%)	1360 (34%)
Unknown indication^b^	4481 (69.8%)	325 (26.2%)	2286 (22%)	226 (23.9%)	1273 (31.8%)
Bipolar disorder	141 (2.2%)	11 (0.9%)	224 (2.2%)	71 (7.5%)	186 (4.6%)
Epilepsy	467 (7.3%)	115 (9.3%)	322 (3.1%)	382 (40.4%)	1027 (25.7%)
Migraine	394 (6.1%)	107 (8.6%)	47 (0.5%)	286 (30.3%)	183 (4.6%)
30 days					
No indication^a^	280 (4.4%)	509 (41%)	5966 (57.4%)	27 (2.9%)	1142 (28.5%)
Unknown indication^b^	4764 (74.3%)	445 (35.9%)	3629 (34.9%)	221 (23.4%)	1420 (35.5%)
Bipolar disorder	233 (3.6%)	14 (1.1%)	328 (3.2%)	73 (7.7%)	197 (4.9%)
Epilepsy	666 (10.4%)	142 (11.4%)	389 (3.7%)	382 (40.4%)	1077 (26.9%)
Migraine	477 (7.4%)	132 (10.6%)	87 (0.8%)	291 (30.8%)	200 (5%)
Anytime					
No indication^a^	0	< 5		< 5	10 (0.2%)
Unknown indication^b^	2281 (35.6%)	565 (45.5%)	6567 (63.2%)	167 (17.7%)	1080 (27%)
Bipolar disorder	941 (14.7%)	57 (4.6%)	1531 (14.7%)	109 (11.5%)	416 (10.4%)
Epilepsy	1877 (29.3%)	276 (22.2%)	1399 (13.5%)	420 (44.4%)	2214 (55.3%)
Migraine	1660 (25.9%)	371 (29.9%)	1120 (10.8%)	382 (40.4%)	553 (13.8%)

^a^ No indication: At index date, the person did not have a record of any condition/diagnosis.

^b^ Unknown indication: None of the specific indications was recorded on index date, but there was a record for any other condition/diagnosis.

Initial daily dose/strength for VPA ranged between a median of 500 and 875 mg/day across databases. Average treatment duration varied substantially, with a median of 50 days in IQVIA Belgium LPD, 82, 98, and 100 days in CPRD GOLD, IPCI, and IQVIA DA Germany, respectively, and 1 year in SIDIAP. Initial dose did not change over the course of the study. However, cumulative annual use decreased in SIDIAP (starting from 2012) and IQVIA DA Germany (starting from 2019), while it remained generally stable for CPRD GOLD, IPCI, and IQVIA LPD Belgium. Drug utilisation also varied by indication, with lower initial dose/strength and shorter treatment duration seen for VPA when used for migraine compared to bipolar disorder and epilepsy in CPRD GOLD, IPCI, and SIDIAP.

## 4 Discussion

This manuscript reports on the first drug utilisation study conducted in the recently established DARWIN EU initiative. We used standardized federated analytics to estimate the incidence and prevalence of VPA use across 6 European countries.

Our analyses showed a decline in VPA treatment initiation in European women of childbearing age. Prevalence was highest in older women. Younger participants had a lower prevalence, which decreased over time. Incidence decreased in all age groups. Alternative therapies with similar indications were studied, and gabapentinoids showed an increase in use during the study period amongst CPRD, ACI VARHA, and SIDIAP.

At the patient level, VPA cumulative dose declined over time in SIDIAP and IQVIA DA Germany, while it remained generally stable in CPRD, IPCI, and IQVIA LPD Belgium. The prevalence of prescriptions of hormonal contraceptives in valproate users seemed to be low. Overall, lower doses and shorter VPA treatment durations were seen for migraine compared to bipolar disorder and epilepsy.

### 4.1 Research in Context

In line with our findings, previous studies assessing the population‐level use of VPA in young women in Europe reported a reduction in the prevalence of VPA use between 2010 and 2020 across multiple European countries [6, 7], with a 37.7% decrease in VPA prevalence in CPRD similar to our study (32.7% from 2010 to 2019). However, while both studies showed a substantial decrease in incidence over time, Abtahi et al. report no significant decrease in new VPA users after the 2018 RMM was implemented compared to the period before [7]. Despite efforts to prevent valproate prescriptions to women of childbearing age, challenges such as the difficulties during clinical management hinder the achievement of this goal.

The findings from our study align with several trends observed in previous studies but also reveal notable differences that emphasize regional and methodological variations: While no significant increase in switching rates from valproates to alternative treatments after the 2018 RMM was seen in the study by Abtahi et al., studies conducted before the 2018 RMM reported variations in antiepileptic prescriptions during pregnancy, hence indicating the possibility of further reductions for valproate use [8, 9]. A study from the UK, Italy, and France among pregnant women observed an increase in the prescription of pregabalin and/or gabapentin between 2007 and 2016, particularly in the UK [8]. Our study found a similar tendency of increased incidence rates of gabapentin and pregabalin, predominantly in the UK and Spain. However, potential risks of pregabalin and gabapentin exposure during pregnancy are not yet well understood. Other studies showed lamotrigine to be the most prescribed antiseizure medication during pregnancy in several countries [8, 9, 10], which seems reassuring given that lamotrigine was associated with a lower risk of teratogenicity [20, 21].

The prevalence of medication use across six databases from 2010 to 2022 highlights significant differences in trends between drugs and regions. Pregabalin shows the highest and most consistent growth, particularly in databases like CPRD GOLD and SIDIAP, where its use increases steadily over time. Gabapentin also shows upward trends in some databases, though less pronounced. In contrast, drugs like carbamazepine and valproate/valpromide remain relatively stable and low in prevalence across all datasets. The observed rise in gabapentinoids (pregabalin and gabapentin) could be linked to their expanded use for neuropathic pain, but also raises concerns about potential overprescription or misuse. Meanwhile, databases like IQVIA GERMANY DA and IPCI show more stable patterns, suggesting steadier prescribing habits. However, there is uncertainty about the safety of their use during pregnancy and while breastfeeding: Previous reports have reported potential risks of malformations associated with pregabalin [22], leading to regulatory measures and changes in product labeling [23, 24, 25]. However, recent studies cast doubt on the association between exposure during pregnancy and major congenital malformations, low birth weight, autism, or other birth defects [26]. Therefore, evidence on the safety of gabapentin during pregnancy is even more limited [27]. In this context, given the recent regulatory modifications, the observed increase in the utilization of gabapentinoids in recent years casts worries, and measures that advocate the prescription of drugs with more robust evidence of safety during pregnancy, such as lamotrigine and levetiracetam [28, 29] where possible, should be strengthened. This variability underscores the need for further investigation into regional and clinical factors influencing these trends, as well as the implications for public health and prescribing guidelines.

Our patient‐level analyses showed high rates of anxiety and depression in people initiating VPA, suggesting the need for extra attention for these conditions in young women with epilepsy and bipolar disorder. Indication of use of VPA was generally not well documented in our electronic health records. Where a relevant diagnosis was previously recorded, migraine and epilepsy were the most common indications, with country‐specific differences owing to for example no authorisation for use in migraine prophylaxis. While the initial VPA dose did not change over the course of the study, the cumulative annual use decreased in SIDIAP and IQVIA DA Germany from 2012 to 2019, respectively. Rates of hormonal contraceptive coverage at the start of valproate treatment were generally low to moderate, and high variation was seen for age groups. The level of prescription of contraceptives was highest in CPRD GOLD, followed by IQVIA Belgium LPD, IPCI, and lowest in SIDIAP and IQVIA Germany DA, with differences potentially due to country‐specific reimbursement strategies and completeness of recordings of contraceptives in the different databases. This fact was reflected in previous studies [7]. In some countries, contraceptives are reimbursed by the healthcare system, which encourages formal prescription and thus increases the likelihood of capture in electronic health records, even in non‐claims databases. In contrast, in countries where contraceptives are frequently purchased over‐the‐counter or are not reimbursed, their use may be under‐recorded.

Use of hormonal contraception varied greatly across age groups, with the highest levels of prescriptions being observed in women between 15 and 39 compared to lower rates in the > 50 and 12–14 year age groups. The age at first valproate (VPA) prescription appears to be notably high. This trend could reflect its predominant use in specific conditions that are more likely to be diagnosed later in life, such as certain psychiatric disorders (bipolar disorder). It may also indicate caution in prescribing VPA to younger populations due to well‐documented safety concerns, such as teratogenic risks and cognitive side effects.

In our study, we observed substantial differences in the incidence of valproate use across data sources, which likely reflect variations in healthcare systems, prescribing practices, and demographic characteristics. Among them, the lowest VPA prevalence appears to be observed in IQVIA Germany (< 0.1%), which is notably lower than the prescribing incidence reported in other studies from the same country [30], such as one citing 0.84/1000 women in 2010 and 0.36/1000 in 2020. However, this discrepancy may be due to methodological differences between databases, and we cannot exclude that it also reflects limitations in the suitability of IQVIA Germany to fully capture valproate prescribing, particularly given the lack of cross‐practice patient identification and the outpatient‐only nature of the data. Similarly, Abtahi et al. seven reported regional disparities in the prevalence of valproate use, with significant declines in Tuscany, Spain, and the UK, but only a non‐significant decline in the Netherlands. These findings resonate with the patterns in our prevalence data, where certain countries and datasets showed sharper declines, highlighting the influence of local regulatory efforts and adherence to risk minimization measures (RMMs).

Regarding incident use, Abtahi et al. found no significant decline following the 2018 RMMs, which aligns with our results showing that incidence rates decreased at a slower pace in some datasets than prevalence. This suggests that while measures to reduce ongoing use may have been effective, preventing new prescriptions remains a challenge across different regions. However, our study demonstrates a slightly sharper decline in incident use in certain datasets compared to the broader trends reported by Abtahi et al., potentially reflecting more recent or localized impacts of interventions.

Another point of comparison is the compliance with contraceptive coverage among women of childbearing age. We found low contraceptive coverage among valproate users across all data sources, ranging from 1.5% in IQVIA DA Germany to 21.9% in CPRD GOLD, with the highest rates observed among younger women (e.g., 42.8% in CPRD GOLD for those aged 20–24). These levels are consistent with the low compliance (< 25%) reported by Abtahi et al., with only modest post‐2018 improvements in the Netherlands.

Our findings complement those of Abtahi et al., particularly regarding regional variability and the challenges in this case, reducing valproate use. These findings align with the regional variability observed in other aspects of valproate use in our study, such as comorbidities. The striking differences in the prevalence of comorbidities across data sources, particularly the higher rates observed in IQVIA Belgium for conditions like COPD, GERD, and hypertension, likely reflect a combination of factors. These differences may be partially explained by the characteristics of the data source, such as the methods used to capture diagnoses or differences in healthcare coding practices, as well as by variations in the underlying population of new valproate users. Specifically, regarding migraine, it stands out that 30% of valproate prescriptions were accompanied by a migraine diagnosis on the same day in one of our databases. This could suggest that valproate is being increasingly prescribed for migraine management in our databases and contrasts with the findings of Abtahi et al., where migraine indication remained steady.

These findings underscore the importance of multidatabase studies using common data models (CDMs), as they enable the harmonization of data across diverse healthcare systems, facilitating the identification of regional differences in drug utilization, comorbidities, and prescribing practices that would otherwise remain obscured in single‐database analyses.

### 4.2 Strengths and Limitations

Our study had strengths and limitations. As for limitations, we acknowledge that a recording of a prescription did not mean that the person took the drug. Therefore, an assumption of actual use and the duration of drug use was made. We assessed indication via a proxy based on pre‐defined conditions recorded on the date of therapy initiation. Therefore, the recording of potential indication might be incomplete. VPA prescriptions in ACI VARHA were only fully collected from 2013/2014 onwards. In Finland, anti‐epileptic drugs other than VPA are only reimbursed if the person was prescribed VPA first. In clinical practice, VPA prescriptions might therefore only be issued to bypass this rule without patients actually starting VPA but directly moving to the next treatment. This prescription practice might increase incidence rates and bias results upwards. In addition, the completeness of recordings of comorbidities used for patient characterisation varied across databases. Although the study population was defined from 2010 to 2022, CPRD GOLD data were only available until 2019 at the time of extraction. While more recent years had been requested, data availability reflected the extraction period. This should be considered when interpreting time trends from this source. Due to the characteristics of the population studied and the lack of a link between women with gestational potential and the outcome of their possible pregnancy, the impact of taking VPA on the outcome of the possible pregnancy could not be evaluated. Also, the approach for calculating incidence rates has limitations. The 30‐day gap criterion may lead to the misclassification of some patients as new users if they resumed treatment after a slightly longer interruption, potentially overestimating incidence rates.

One key limitation of this study relates to the heterogeneous availability and quality of data on prescribed dose and treatment duration across databases. While some sources provided explicit information on these variables, others required assumptions to approximate daily dose or treatment length based on product strength and expected daily dosing. These estimations introduce uncertainty and may lead to misclassification of initial dose or treatment duration, particularly in indications where dosing varies widely (e.g., epilepsy vs. migraine). Additionally, for databases like ACI VARHA, inpatient prescriptions—which represented the majority—lacked dose and duration data altogether, limiting the completeness of treatment characterization. These limitations should be taken into account when interpreting dose‐related findings.

Finally, the observed differences in the prevalence of valproate and alternative medication use across regions may reflect not only true variations in clinical practice but also differences in data capture, completeness, and healthcare system organization. For example, the lowest prevalence was observed in IQVIA Germany (< 0.1%), which contrasts with higher prescribing incidence in other countries but is also reported in previous national studies. This discrepancy may be partially explained by the characteristics of the IQVIA Germany database, which does not allow cross‐practice patient identification due to data protection constraints. These factors may have led to an underestimation of medication use and the potential for duplicate patient counts. More broadly, there were marked differences in data completeness across the six participating databases. For example, while SIDIAP (Spain) captures both prescription and dispensation records from a large primary care population, and ACI VARHA (Finland) includes both inpatient and outpatient specialist prescriptions, information on dose and duration is missing for inpatient prescriptions. In contrast, CPRD GOLD (UK) and IPCI (Netherlands) are based solely on primary care prescription data, while IQVIA LPD Belgium includes both GP and outpatient specialist care prescriptions. These differences in setting, drug capture (prescription vs. dispensation), and population coverage influence the degree of completeness and comparability across databases, and should be considered when interpreting prevalence estimates.

In addition, differences in prescribing practices, medication adherence, and access to healthcare across countries may also affect prevalence estimates and should be considered when interpreting observed trends. It is important to note that our study was exploratory in nature and aimed to assess the feasibility and analytical capacity of a distributed data network across multiple countries. The selection of data partners was based on a balance between having robust denominator populations (typically in primary care datasets) and access to information on secondary care prescribing and dosage. Future studies using more integrated or linkable data sources may help to overcome some of the limitations identified here.

We consider the use of standardized analyses and high‐quality databases previously mapped to the OMOP common data model particular strength. For all databases, comprehensive quality checks were conducted at the stage of onboarding databases to the DARWIN EU network. Upon request of the EMA, we included 6 databases from all across Europe, providing good geographical representation. The inclusion of many participants boosts the statistical power and permits more precise and generalizable results. Databases only contributed to those analyses where their data was deemed of adequate quality and granularity. All analytical packages used for this study were thoroughly tested and are publicly available on CRAN. As these databases were readily available and study code was leveraging pre‐tested analytical pipelines, we were able to conduct the study within 4 months from approval of the protocol by the EMA (28th September 2022) to acceptance of the study report on the 6th of February 2023. This showcases the potential to generate rapid evidence for regulatory decision making without compromising scientific quality and rigour.

### 4.3 Implications and Conclusion

The study analyzed women of childbearing age from six European countries across diverse healthcare systems (primary care in IPCI, SIDIAP, and CPRD; secondary care in ACI VARHA, IQVIA Germany DA, and IQVIA Belgium LPD). While the findings are representative of the study populations within each country, they help us understand what might happen in the general population. The results showed a decline in incident VPA use among women of childbearing age over time, with a sharper decrease in younger age groups. Prevalence initially dropped during the early years of the study and then stabilized at lower levels. Hormonal contraceptive prescriptions were generally low and varied widely between databases, likely influenced by country‐specific reimbursement policies and the completeness of prescription records. Although initial doses of VPA remained consistent, cumulative annual use decreased over time in SIDIAP and, after 2019, in IQVIA Germany DA. Further research is needed to understand the factors influencing these trends, particularly the registered low levels of contraceptive use and potential gaps in prescribing practices to minimize risks in this population.

### 4.4 Plain Language Summary

This study explored how the use of the epilepsy drug valproate (VPA) and its alternatives has changed among young women in Europe over the past decade. VPA is effective for treating epilepsy but can cause serious birth defects, so its use is limited in women of childbearing age. We analyzed health records from six European countries, covering the years 2010 to 2022, to understand trends in VPA use and to see how often alternative treatments, like pregabalin and gabapentin, were prescribed instead. Our study included millions of women, and we found that the number of young women starting VPA treatment has declined since 2015. At the same time, the use of alternative medications has increased. We also observed that women who began using VPA were typically in their early 40s and that few of them were using hormonal contraceptives. This study highlights the importance of ongoing monitoring to ensure the safety of women who need epilepsy treatment. Our findings also show how quickly and effectively data from multiple countries can be used to support health decisions and improve patient safety across Europe.

## Author Contributions

A.P.‐U. and A.M.J. led the conceptualisation of the study with contributions from M.C., E.B., and D.P.‐A. M.C. developed the code for statistical analyses with methodological advice and contributions from E.B., Y.G., and M.D. M.C., T.D.‐S., E.F., J.T.B., and T.K. executed study code in the respective databases. A.P.‐U., A.M.J., C.R., S.S., K.V., D.P.‐A, and L.B. clinically interpreted the results. L.B. wrote the first draft of the manuscript. All authors read, contributed to, and approved the last version of the manuscript. A.P.‐U. and A.M.J. obtained the funding for this research. T.D.‐S. and C.R. had access to and verified the Information System for Research in Primary Care (SIDIAP) data; M.C. and A.P.‐U. had access to and verified the Clinical Practice Research Datalink (CPRD GOLD) data. E.F. and K.V. had access to and verified the IPCI database. T.K. and E.K. had access to and verified the ACI VARHA dataset. A.P.‐U. and A.M.J. were responsible for the decision to submit for publication.

## Ethics Statement

The study was approved by the CPRD's Research Data Governance Process, Protocol No 22_002336, the Clinical Research Ethics Committee of Fundació Institut Universitari per a la recerca a l'Atenció Primària de Salut Jordi Gol i Gurina (IDIAPJGol) (approval number 22(252‐EOm)), the IPCI Review Board (registration no. 11/2022). Approval from an Ethics Committee was not required for the use of ACI VAHRA, IQIVA DA Germany, and IQVIA LPD Belgium for this study.

## Conflicts of Interest

For this project, a data partner's role was only to execute code at their data source and data partners did not have an investigator role. The manuscript received support from the European Medicines Agency through the DARWIN Coordination Center. The authors declare no conflicts of interest related to this study. The authors report the following conflicts of interest not related to this study: K.V. works for a research group that, in the past, received unconditional research grants from UCB, Chiesi, Amgen, and J&J. J.T.B. worked on a project for a pharmaceutical client that explored the use of painkillers in the context of COVID. This project had been completed by the start of 2022, and opioids were not examined in this study. D.P.‐A. department has received grant/s from Amgen, Chiesi‐Taylor, Lilly, Janssen, Novartis, and UCB Biopharma. His research group has received consultancy fees from Astra Zeneca and UCB Biopharma. Amgen, Astellas, Janssen, Synapse Management Partners, and UCB Biopharma have funded or supported training programmes organised by DPA's department.

## Data Availability

CPRD GOLD: CPRD GOLD data were obtained under the CPRD multi‐study license held by the University of Oxford after Research Data Governance (RDG) approval. Direct data sharing is not allowed. SIDIAP: In accordance with current European and national law, the data used in this study is only available for the researchers participating in this study. Thus, we are not allowed to distribute or make publicly available the data to other parties. However, researchers from public institutions can request data from SIDIAP if they comply with certain requirements. Further information is available online (https://www.sidiap.org/index.php/menu‐solicitudesen/application‐proccedure) or by contacting SIDIAP (sidiap@idiapjgol.org). IPCI: Almost all studies using IPCI data concern retrospective research of observational data, and as such are not subject to the Medical Research Involving Human Subjects Act (WMO) and do not require approval from a medical research ethical committee. If additional patient data have to be collected, the protocol is sent to an accredited medical research ethical committee for review. Before data are transferred to the central database, data are pseudonymized. The data are stored on an isolated network without an internet connection. Within this network, there are separate layers to distinguish access for data supply to GPs, data coding, and data analysis. Data access is only possible at authorized computers in secured rooms and after authorized user access. Employees working with the IPCI and external researchers must sign a declaration of confidentiality. Only aggregated data are allowed to leave the secured environment. The study protocol was approved by the review board of IPCI on 22nd June 2023. IQVIA: IQVIA Germany DA and IQVIA LPD Belgium in the OMOP format are commercially available databases that can be purchased and licensed by any researcher. The collection and de‐identification of these data assets is a process that is commercial intellectual property and not privileged to the data licensees and the co‐authors on this study. Licensees of these data have signed Data Use Agreements with the data vendors which detail the usage protocols for running retrospective research on these databases. All analyses performed in this study were in accordance with Data Use Agreement terms as specified by the data owners. As these data are deemed commercial assets, there is no Institutional Review Board applicable to the usage and dissemination of these result sets or required registration of the protocol with additional ethics oversight. Compliance with Data Use Agreement terms, which stipulate how these data can be used and for what purpose, is sufficient for the licensing commercial entities. Further inquiry related to the governance oversight of these assets can be made with the respective commercial entity: IQVIA (iqvia.com). At no point in the course of this study were the authors of this study exposed to identified patient‐level data. All result sets represent aggregate, de‐identified data that are represented at a minimum cell size of 5 to reduce potential for re‐identification. ACI VARHA: The data covers the patient register at the Wellbeing services county of Southwest Finland (VARHA) (formerly, Hospital District of Southwest Finland—HDSF). It covers the public specialist health care and most emergency health care in the area of Southwest Finland. The data is utilized for scientific research under Finnish legislation (The Act on Secondary Use of Health and Social Data) and requires a data permit; thus, it isn't publicly available nor possible to share or distribute without.
